# A Psychometric Study of a Spanish Version of the Negative Acts Questionnaire-Revised: Confirmatory Factor Analysis

**DOI:** 10.3389/fpsyg.2020.01856

**Published:** 2020-08-12

**Authors:** Víctor Dujo López, David González Trijueque, José L. Graña Gómez, José M. Andreu Rodríguez

**Affiliations:** ^1^Department of Personality, Assessment and Clinical Psychology I, Faculty of Psychology, Complutense University of Madrid, Madrid, Spain; ^2^Department of Clinical Psychology, Faculty of Psychology and Educational Sciences, Francisco de Vitoria University, Madrid, Spain

**Keywords:** workplace harassment, negative acts questionnaire, assessment, psychometric analysis, psychosocial stressor

## Abstract

**Background:**

The NAQ (Negative Acts Questionnaire) has been widely used in more than 40 countries to measure the mobbing phenomenon. This research aims to present a psychometric study based on the Spanish version of the NAQ-R carried out by González-Trijueque and Graña (2013). As opposed to the original scale, this sample contains 23 items and a three-dimension model (personal bullying, work-related bullying, and physically intimidating forms of bullying).

**Methods:**

We used a heterogeneous occupational sample of 2,538 Spanish employees to analyze internal consistency and concurrent validity. In addition, a Confirmatory Factor Analysis was carried out based on the GLS (generalized least squares) method.

**Results:**

Results showed high internal reliability (Cronbach’s alpha = 0.91) and high correlations containing clinical symptoms, burn-out indicators, and coping resources. The Confirmatory Factor Analysis developed upon the heterogeneous occupational sample validates the three-dimension structure in the Spanish version of the NAQ-R aimed to measure harassment behaviors at the workplace.

**Conclusion:**

The NAQ-R reaffirms its validity and reliability as a measure for mobbing-related behaviors. Hence, the scale may become a useful tool in research and forensic practice.

## Introduction

Based on the epidemiological data, workplace harassment highlights the devastating impact at both human and organizational levels (EUROFOUND, 2017; Rodríguez-Muñoz et al., 2017; Hogh et al., 2019; El Ghaziri et al., 2020). From the perspective of forensic psychology, this paper validates one of the main instruments to measure mobbing with practical and clear implications for the development of forensic psychology in the justice administration.

From a psycho-legal perspective, the legal definition of mobbing has drawn on the doctrine of case law, psychology, and psychiatry literature. Therefore, relying on instruments with good psychometric properties for the measurement of mobbing, experts are guaranteed with a suitable tool, not only in forensic and psychological terms but also for Legal Psychology with regard to establishing psychological constructs in law courts (González-Trijueque and Graña, 2013).

We are looking toward a growing reality that in the last years has become a socio-occupational phenomenon and it turned into a topic of international interest as a result of the impact at both personal and socio-economic levels (Einarsen et al., 2011; EUROFOUND, 2015, 2017). We are not facing an isolated event but a process where an employee or employees experience different types of negative behaviors from co-workers. Within this persistent and long-term exposure may exist a power role between the formal and informal pair. Assuming the previously mentioned, this also may present a significant risk to the employee’s health (Fidalgo et al., 2009).

Mobbing prevalence data is heterogeneous, varying from 2 to 17% (Nielsen et al., 2011). Figures gathered from the Spanish working population showed a prevalence rate of 13.2% (Barómetro Cisneros, 2009). The Sixth European Working Conditions Survey (EUROFOUND, 2017) displays that at least 5% of the European workforce had been exposed to harassment behaviors within 12 months prior to the study. The health sector reported the greatest intensity of workers exposed to these behaviors. Since 2010, there has been a growing trend that is explained by the economic crisis with the subsequent socio-labor impact (EUROFOUND, 2015).

In addition to what we already know about this phenomenon, it is necessary to specify that while negative behaviors are key in conceptualizing mobbing, its essence is significant not only for the heterogeneous nature of behaviors but also in the tenacity and systematicity of experience (Einarsen et al., 2003; Trépanier et al., 2015) as the long-term exposure tends to wear down the coping strategies of the victims. It is therefore important to note that the harassment pattering occurs mostly when a power imbalance exists as a core feature of mobbing. The situation limits victims’ response and they can find difficulty in defending themselves. The imbalance of power often can come from a formal power structure of the organizational context (Hutchinson et al., 2006; Park et al., 2018) or may also be informal, based on experience, knowledge or access to support from influential persons (Einarsen et al., 2003).

Since the eighties, numerous methods were developed to measure harassment both qualitatively and quantitatively. Questionnaires remain the most used methods because they are very quick and easy to use (Leymann, 1990). Mobbing is a complex phenomenon with various facets and its measurement is a demanding task (Cowie et al., 2002; Einarsen et al., 2003). Three measurement methods can be applied in the assessment of mobbing. The first one asks the participants whether they identify with the definition of mobbing. In the second one, the operational criterion method is carried out through questionnaires, and the third method combines the previous two considered to be the best approach for the assessment of mobbing (Mikkelsen and Einarsen, 2001; Salin, 2001; Nielsen et al., 2011).

Within the subjective approach, the person being assessed is required to reply to a yes/no question whether they consider themselves as mobbing victims. The main problem of this approach is that subjective experience of harassment refers to targets’ perceptions of their experience and cultural values, which creates interpersonal variability (Niedl, 1995).

The objective approach, based on Leymann’s work, consists of a set of items measuring employee’s exposure to harassment behaviors. Harassment-related items were excluded to avoid victimization and socio-cultural biases (Giorgi et al., 2011; León-Pérez et al., 2019a). This method presents methodological issues such as the definition of an arbitrary cutoff point and heterogeneity among harassment behaviors considering that each questionnaire differs in terms of the number of items and topic, and the important variability within the harassment behaviors. This implies that a low-level exposure could be severe depending on each negative act (Notelaers et al., 2006).

Among the most important research instruments on the behavioral report method, there is the Leymann (1990) LIPT-60 (Leymann Inventory of Psychological Terrorization) and the NAQ-R (Einarsen and Hoel, 2001; Einarsen et al., 2009; Nolfe et al., 2012). At the national (Spanish) level, there are several different versions of the NAQ-R: the NAQ-RE (Sáez et al., 2003); two reduced versions, the NAQ-14 (Moreno-Jiménez et al., 2007) and the S-NAQ (León-Pérez et al., 2019b) and the NAQ-P, and an adapted version for perpetrators (Escartín et al., 2012).

At the international level, the NAQ (Einarsen and Raknes, 1997) is one of the most widely used instruments to evaluate any form of negative behavior (Nielsen et al., 2009, 2011). The original scale involved 24 items measuring exposure within the last 6 months without being identified as harassment using a five-item Likert scale (1, Never; 2, Now and Then; 3, Monthly; 4, Weekly; 5, Daily). By taking this into consideration, non-harassment-related behaviors are to avoid respondents to previously act in a non-judgmental way with regard to their role as victims, thus achieving more impartial results. Once the responses are gathered, a definition of mobbing is presented to the participants, allowing them to identify whether they experienced it or not (González-Trijueque and Graña, 2013).

Internal consistency of the NAQ has shown high internal consistency as measured by Cronbach’s alpha, which was 0.87 for personal harassment and 0.81 for work-related harassment (Einarsen and Hoel, 2001). Although the original scale showed high internal consistency containing items with good validity and evidence of good construct validity, the scale also had some constraints. Its validity was tested within a limited Scandinavian cultural context, so additional issues were found in the mainstreaming of the results as well as in its factor structure. Hence, a revised scale was needed. The NAQ-R was developed with the aim of achieving a greater cross-cultural validity while developing and refining the items based on the original scale (Einarsen et al., 2009).

All items in the NAQ-R scale are developed in behavioral terms with no reference to bullying or harassment. Cronbach’s alpha for the 22 items in the NAQ-R indicates a satisfactory internal consistency. In order to test the underlying dimension of the NAQ-R, a confirmatory approach with a positive outcome was pursued (Einarsen et al., 2009). Following the original NAQ, the NAQ-R is a 22-item scale containing an additional item used to describe behavioral terms without referring to mobbing definition, which comprises a reliable and valid measure of exposure to mobbing (Einarsen et al., 2009).

The NAQ-R has been validated within numerous international studies (Nielsen et al., 2011; Charilaos et al., 2015), indicating a satisfactory transcultural construct validity (Table 1; González-Trijueque and Graña, 2009). When conducting the in-depth study, based on its conceptualization, it can be used as a one-dimensional model (mobbing as one-factor); bidimensional (mobbing and personal harassment assessment); or three-dimensional (added to the previous factors, physical and/or sexual harassment). The results of the three models show a high correlation: 0.96 for personal and work-related harassment; 0.89 for work-related harassment and intimidation; and 0.83 for personal harassment and intimidation (Einarsen et al., 2009).

**TABLE 1:** Cross-cultural validation of the NAQ-R.

Study	Results
NAQ-R (Einarsen et al., 2009)	Internal stability with three underlying factors.
	0.96 for work-related and person-related bullying
	0.89 for work-related bullying and intimidation
	0.83 for person-related and intimidation
Validation of the NAQ-R in Norway (Nielsen et al., 2009)	Internal reliability: Cronbach’s alpha was between 0.88 and 0.90
Validation of the NAQ-R in Japan (Tsuno et al., 2010)	Internal reliability: Cronbach’s alpha was between 0.91 and 0.95 for both men and women.
	High concurrent validity on LIPT-60
Psychometric properties of the NAQ-R in India (Rai and Agarwal, 2017)	Internal reliability: Cronbach’s alpha was 0.97.
	Two-factor model:
	Factor I: work-related bullying: 0.89
	Factor II: person-related bullying: 0.97
	Values for the convergent and discriminant validity were statistically significant (<0.50)
Validation of the NAQ-R in Brazil (Maciel and Gonçalves, 2008)	Reliability: Cronbach’s alpha for internal consistency was 0.90
Psychometric properties of the NAQ-R in Brazil (Silva et al., 2017)	Internal validity for men
	Factor I: work-related bullying: 0.76
	Factor II: person-related bullying: 0.78
	Internal validity for women
	Factor I: 0.83
Validation of the NAQ-R in the Czech Republic (Cakirpaloglu et al., 2017)	Internal reliability: Cronbach’s alpha was 0.94
Validation of the NAQ-R in Korea (Lee et al., 2016)	Three-factor version
	Cronbach’s alpha was 0.93
Psychometric properties of the NAQ-R in Venezuela (Millán et al., 2016)	Internal reliability: Cronbach’s alpha was 0.95
Psychometric properties of the NAQ-R in Greece (Kakoulakis et al., 2015)	Internal reliability: Cronbach’s alpha was 0.915
Italian version of the NAQ-R (Giorgi et al., 2011)	Internal reliability: Cronbach’s alpha was 0.91
	Two-dimension model:
	0.91 for personal bullying
	0.70 for work-related bullying
Serbian version of the NAQ-R (Vukeliæ et al., 2015)	Internal reliability: Cronbach’s alpha was 0.96
Spanish version of the NAQ-R (González-Trijueque and Graña, 2013)	Internal reliability: Cronbach’s alpha was 0.93
	Three-factor model
	Psychological harassment: 0.93
	Work-related harassment: 0.81
	Physical abuse: 0.66
Validation of the NAQ-R in Estonia (Tambur and Vadi, 2009)	Internal reliability: Cronbach’s alpha was 0.91

González-Trijueque and Graña (2013) added one sexual harassment-related item to the Spanish version of the NAQ-R, thus containing 23 items in total. Based on the review of the literature and with the objective to define a three-dimension model measuring all the existing violence subtypes, the authors deemed it necessary to make a distinction between personal, workplace, physical, and sexual harassment. The results reported a high internal consistency with significant reliability in regard to the Cronbach’s alpha, which was 0.93 (exceeding the 0.90 of the original scale). In this sense, when analyzing the internal consistency of each subscale, the personal-related harassment was the most accurate (0.93) followed by work-related harassment (0.81) and physical abuse (0.66). With reference to the concurrent validity, there is a positive and relevant correlation between the NAQ-R scores and both psychopathological symptoms and burnout. Therefore, the questionnaire is a reliable and valid instrument when assessing mobbing.

A revised scale is needed as the previous Spanish scale (González-Trijueque and Graña, 2013) did not include a confirmatory approach to successfully replicate the factor structure.

## Objectives And Hypotheses

Objective #1: Psychometric study of a Spanish version of the NAQ-R (reliability and validity).

Objective #2: To analyze the concurrent validity between the NAQ-R and clinical symptoms (BSI), burn-out symptoms (MBI), and coping strategies (COPE).

Objective #3: To assess the use of the NAQ-R in the forensic framework as a future research direction.

Hypothesis #1: The NAQ-R is a suitable instrument for measuring psychological harassment at work. The instrument will adequately discriminate between harassed and non-harassed victims.

Hypothesis #2: Victims will show more clinical and burnout related symptoms than the non-harassed group of active employees.

Hypothesis #3: The coping strategies of the victims will depend on the type of harassment they were exposed to (psychological, work-related and physical).

## Materials and Methods

### Participants

For this study, we used a heterogeneous occupational sample including 2,538 employees from the Community of Madrid as the only inclusion criteria. The sampled covered the working population included in the census of the Madrid Region. Data were collected from the National Statistical Institute.

### Measurements

Socio-demographic data and socio-occupational questionnaires were included *ad hoc* for this study and additional instruments were used: the Spanish version of the NAQ-R (González-Trijueque and Graña, 2013), the Brief Symptom Inventory (BSI) (Derogatis, 1975, 1993, Spanish adapted version of Aragón et al., 2000), the Maslach Burnout Inventory (MBI) (Maslach and Jackson, 1986, 1997, published in Spanish by TEA, 1997), and the Brief COPE questionnaire (Carver, 1997, Spanish adapted version of Crespo and López, 2003). The NAQ-R is the instrument chosen for this study as it is the most widely used measurement questionnaire for the study of the prevalence of psychological harassment at work and has been validated across many countries and instruments (Notelaers and Einarsen, 2013).

The BSI evaluates clinically relevant psychological symptoms measuring distress and psychiatric disorders covering the following nine symptom dimensions: Psychoticism (Psy), Paranoid Ideation (Par), Phobic Anxiety (Pho), Hostility (Hos), Anxiety (Ans), Depression (Dep), Interpersonal Sensitivity (Int), Obsession–Compulsion (Obs), and Somatization (Som); and the global indices of distress: Global Severity Index (GSI), Positive Symptom Distress Index (PSDI), and Positive Symptom Total (PST). In addition to the total score of the scale, the MBI measures Personal Accomplishment (PA), Depersonalization (DP), and Emotional Exhaustion (EE). In the present study, the shortened version of the Brief COPE was used in order to compile the results under the different subscales: (A) Active coping, (B) Planning, (C) Use of instrumental support, (D) Use of emotional support, (E) Religion, (F) Acceptance, (G) Denial, (H) Substance use, (I) Humor, (J) Self-distraction, (K) Behavioral disengagement, (L) Venting, (M) Positive reframing, and (N) Self-blame.

### Methodology

The stratified sample was collected thanks to 250 student assistants from the Faculty of Psychology at the Complutense University who were properly trained in mobbing research and data collection. In addition, regular meetings with all the students have taken place to improve their knowledge and skills in the context of mobbing and data collection procedures. The task entrusted to each student was to distribute voluntarily and confidentially 16 questionnaires to the working population in their community, thus fulfilling the criteria inclusion. Based on the census of each geographical area, all 250 students were allocated to different areas within the Community of Madrid. After analyzing a total of 4,000 questionnaires distributed, the final number of valid respondents was 2,538. Eventually, a total of 1,462 questionnaires were excluded as clearly not meeting the specific criterion due to inconsistent or random answers. For the purpose of applying the latter criterion, four items of similar content were included in the set of questions specifically developed for this study.

The objective was to take a representative sample of the active working population. To this end, the working population is taken as a reference based on the census of the Region of Madrid. The data from the census was obtained from the National Institute of Statistics (“Revision of the population projections in the Community of Madrid 1996–2011”); these data were confidential. To achieve the objectives of the study, all 250 research assistants were assigned to different areas of the Community of Madrid considering the population census and the following geographical areas in order to obtain the study sample:

a) Center of Madrid 51% (126 research assistants)
b) Northern metropolitan area 5% (12 research assistants)
c) Western metropolitan area 5% (14 research assistants)
d) Southern metropolitan area 18% (45 research assistants)
e) Eastern metropolitan area 8% (21 research assistants)
f) Peripheral metropolitan area 13% (32 research assistants).

Each research assistant is assigned a census. The assistant contacts the participants by phone and explains how they are required to give their consent and how to proceed in order to send the protocols anonymously to a post office box created specifically for this purpose. Participating in the study is voluntary and confidential. The protocols are anonymous and contain simple instructions to familiarize respondents with and facilitate the entire process. The steps to be followed for sending and receiving the protocols are as follows:

1. Students contact the candidate by phone.
2. They are given all the information related to the study and information concerning confidentiality and personal data.
3. Once the candidate agrees to participate (then becomes a participant), he/she is sent an envelope containing the protocol to be completed and a consent form including the same information provided in writing.
4. During the whole process, participants can contact their reference research assistant (each student has 16 participants under his/her charge).
5. Once the participant has filled in the form, he or she will send it anonymously to the postal address provided.

### Statistical Analysis

SPSS (19.0 version) was used to gather statistical analysis in order to examine internal consistency and concurrent validity. Additionally, the coefficient of reliability of Cronbach’s alpha was employed for the main scale and the three subscales. Pearson’s bivariate correlations determined the concurrent validity within symptoms, burnout indicators, and coping. The multivariate kurtosis pointed to the non-normality of the obtained data (Mardia coefficient = 735.4). Consequently, Generalized Least Squares (GLS) was used as estimation method.

## Results

### Descriptive Statistical Analysis

Participants in the sample (Table 2) reported a higher perception of harassment during the last 6 months with regard to performing tasks below their level of competence (item 3:1.93), someone withholding information (item 1:1.71), and unmanageable workload (item 21:2.00). Behaviors showing lower scores are related to intimidating actions item 9 (1.71); item 20 (1.18) and physical and sexual-related behaviors: item 22 (1.06) and (1.07).

**TABLE 2:** Statistical description of data.

	Mean (X)	Standard deviation (SD)
1. Someone withholding information which affects your performance	1.71	0.94
2. Being humiliated or ridiculed in connection with your work	1.34	0.67
3. Being ordered to do work below your level of competence	1.93	1.07
4. Having key areas of responsibility replaced with more trivial or unpleasant tasks	1.59	0.97
5. Spreading of gossip and rumors about you	1.46	0.77
6. Being ignored, excluded or given the silent treatment	1.37	0.76
7. Being insulted or having offensive remarks made about your person, attitudes or your private life	1.29	0.64
8. Being shouted at or being the target of spontaneous anger	1.58	0.78
9. Intimidating behaviors such as finger-pointing, invasion of personal space, shoving, blocking your way, etc.	1.17	0.54
10. Hints or signals from others that you should quit your job	1.27	0.67
11. Repeated reminders of your errors or mistakes	1.43	0.76
12. Being ignored or facing a hostile reaction when you approach	1.38	0.69
13. Persistent criticism of your errors or mistakes	1.35	0.71
14. Having your opinions ignored	1.66	0.83
15. Practical jokes carried out by people you don’t get along with	1.27	0.61
16. Being given tasks with unreasonable deadlines	1.40	0.76
17. Having allegations made against you	1.48	0.70
18. Excessive monitoring of your work	1.69	1.02
19. Pressure not to claim something to which by right you are entitled	1.34	0.75
20. Being the subject of excessive teasing and sarcasm	1.18	0.52
21. Being exposed to an unmanageable workload	2.00	1.14
22. Threats of violence or physical abuse or actual abuse	1.06	0.36
23. Threats of sexual harassment at the workplace	1.07	0.39
NAQ-R (Negative Acts Questionnaire—Revised)	31.9	9.92

*N = 2,538, Range: 1–5.*

A sample of multi-occupational workers among different sectors is used to obtain representative and widespread results and to estimate the prevalence of harassment within the sample. The industries included in the sample are health, education, administration, financial, and business services, communications, transport, hospitality, trade, food and textiles, construction, agriculture, livestock, and fishing, mining, energy chemical, and metal industries, electrical equipment, and vehicles.

Using overall samples of employees and avoiding targeting strictly workplace harassment victims (in forensic and clinical samples) comes from the need to assess whether the questionnaire discriminates between harassed and non-harassed. Also, because when analyzing the correlation with clinical symptoms, vulnerability features are more usual in studies involving clinical samples rather than using multi-occupational samples of active workers (Lind et al., 2009).

Of the total sample, 97.8% of the 2,538 employees are Spanish. A total of 55.4% are females and 44.6% are males. The mean age for the total sample was 33.87 years (34.92 years for men and 33 for females). A total of 76.6% participants work within the public sector and the remaining 23.4% work in the private sector. In terms of the socio-economic level, a total of 86.1% middle-class participants with higher education (43.8%) and single employees (59.9%) prevail.

Regarding the perception of being a victim of workplace harassment, the 9% of respondents reported being a victim, of whom 2% reported more frequent exposure and 7% reported experiencing workplace harassment occasionally. The most common type of mobbing is horizontal harassment inflicted by peers and superiors (4.1%) followed by bossing (3.5%), which is a type of harassment coming in an upward vertical direction. A total of 81.6% of participants stated not having received previous psychological counseling and only the 5.5% stated receiving current psychological support.

### Reliability

In the first place, we had to determine the level of reliability and its factors shown by Cronbach’s alpha coefficient in Table 3. Reliability data underline good scores for both full scale (0.91) and psychological distress scale (0.90). Scales for work-related harassment (0.75) and physical intimidation (0.56) are discrete and less accurate. The average variance extracted for these scales were 0.35, 0.35, and 0.37, respectively. Besides, the composite reliability was satisfactory for psychological distress scale (0.93) and work-related harassment (0.74) but less accurate for physical intimidation (0.57).

**TABLE 3:** Internal consistency reliability.

	Cronbach’s alpha
Total harassment (items = 23)	0.91
Psychological harassment (items = 15)	0.90
Work-related harassment (items = 6)	0.75
Physical abuse (items = 2)	0.56

### Concurrent Validity Among Dimensions of the NAQ-R and BSI

To measure concurrent validity, we took as reference the common criteria used in the literature to assess harassment, reliability, and validity. As shown in Table 4, the NAQ-R and the BSI were positively correlated with all the scales and significantly with psychological harassment and interpersonal sensitivity, depression, anxiety, hostility, and paranoid ideation.

**TABLE 4:** Correlations among dimensions of the NAQ-R and BSI.

	Som.	Obs.	Int.	Dep.	Ans.	Hos.	Pho.	Par.	Psy.	GSI	Total
Psychological harassment	0.32**	0.37**	0.43**	0.42**	0.40**	0.42**	0.32**	0.54**	0.37**	0.49**	0.48**
Work-related harassment	0.29**	0.35**	0.33**	0.36**	0.36**	0.38**	0.24**	0.46**	0.28**	0.42**	0.41**
Physical abuse	0.14**	0.10**	0.13**	0.15**	0.12**	0.16**	0.15**	0.15**	0.16**	0.17**	0.17**
NAQ-R Total	0.34**	0.40**	0.43**	0.44**	0.43**	0.45**	0.32**	0.56**	0.37**	0.51**	0.51**

**p < 0.01, *N* = 2,538.

### Concurrent Validity Among Dimensions of the NAQ-R and COPE

The results show a great variety of coping strategies, especially at the workplace and personal harassment. Results in Table 5 suggest that in situations where stressors are more distinguishable (e.g., intimidation behaviors or threats of physical or sexual abuse), coping resources are more discriminant but also tend to passiveness. Hence, correlations for coping resources, seeking instrumental and emotional support, planning, positive reframing, and acceptance are non-significant. However, if the existing behaviors are general or less recognizable by the victim, a wider spectrum is considered; thus, all scores are significant.

**TABLE 5:** Correlations among dimensions of the NAQ-R and COPE.

	Self-distr.	Act. c.	Den.	Subst.	Emot.	Inst.	Disen.	Ven	Refr	Plan	Hum	Accep	Rel.	Self blam.	Total
P.H.	0.22**	0.16**	0.24**	0.17**	0.16**	0.17**	0.26**	0.25**	0.14**	0.20**	0.16**	0.16**	0.14**	0.24**	0.3**
W.H.	0.15**	0.17**	0.18**	0.14**	0.18**	0.17**	0.18**	0.21**	0.16**	0.20**	0.16**	0.14**	0.03	0.18**	0.26**
Ph.A.	0.4**	0.15	0.11**	0.16**	0.02	0.02	0.10**	0.05*	-0.003	0.17	0.70**	0.00	0.05**	0.03**	0.07
NAQ-R	0.21**	0.18**	0.24**	0.18**	0.18**	0.18**	0.25**	0.26**	0.16**	0.21**	0.18**	0.16**	0.11**	0.24**	0.31**

**p < 0.01, *N* = 2,538.

### Concurrent Validity Among Dimensions of the NAQ-R and MBI

Following Pearson’s bivariate correlations, the aim is to find correlations between burnout and mobbing indicators with significant results in terms of workplace harassment and emotional exhaustion as shown in Table 6.

**TABLE 6:** Correlations among dimensions of the NAQ-R and MBI.

	Emotional exhaustion	Depersonalization	Personal accomplishment	Burnout
Psychological harassment	0.43**	0.37**	0.15**	0.45**
Work-related harassment	0.48**	0.33**	0.12**	0.45**
Physical abuse	0.10**	0.16**	0.09**	0.16**
NAQ total	0.49**	0.39**	0.16**	0.49**

**p < 0.01, *N* = 2,538.

### Confirmatory Factor Analysis

**FIGURE 1:**
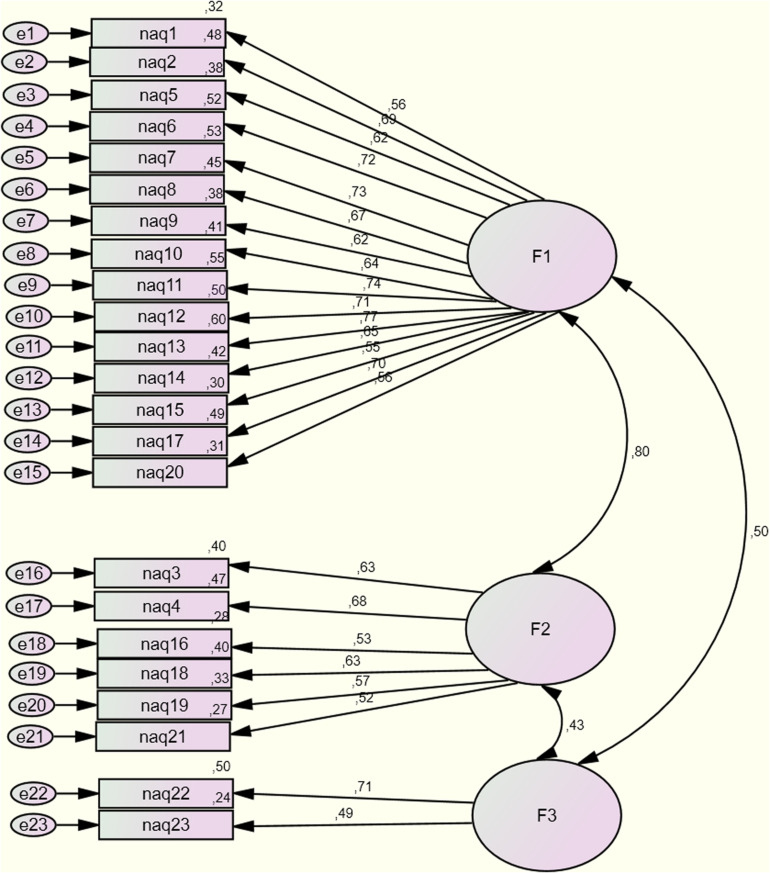
Confirmatory factor analysis of the three-factor model (personal harassment, work-related harassment, physical abuse and/or sexual harassment).

The confirmatory factor analysis based on 23 items showed suitable fit indices: root mean square residual (RMR) = 0.04; adjusted goodness of fit index (AGFI) = 0.93; root mean square error of approximation (RMSEA) = 0.05 and Tucker–Lewis fit index (TLI) = 0.90 (Table 7). Based on the Spanish version of the three-dimension model, the factor-structure loadings exceed 0.35 and the variance explained for each item exceeds 0.20. All correlations among the three factors are significant (*p* < 0.05) and higher scores are associated with personal and work-related harassment (Figure 1).

**TABLE 7:** Confirmatory factor analysis: comparison between three models.

Indices	Three-factor model (figure 1)	One-factor model (figure 2)	General factor model including a three-factor model (figure 3)
RMR	0.04	0.05	0.05
AGFI	0.93	0.90	0.89
RMSEA	0.05	0.06	0.06
TLI	0.90	0.89	0.89

*RMR, root mean square residual; AGFI, adjusted goodness of fit index; RMSEA, root mean square error of approximation; TLI, Tucker–Lewis fit index.*

Nevertheless, in order to verify the three-factor scale, the model was compared with different versions of the NAQ-R. We tested a one-factor model and a general factor solution that groups together a three-factor model. The three-factor model fits the data best as shown in the following table. Higher scores than 0.90 on the AGFI and TLI indicate valid goodness of fit, whereas scores equal to or lower than 0.05 on the RMR and RMSEA show a good fit for this model (Byrne, 2001). The results show that the three-factor model fits the data better: RMR = 0.04, AGFI = 0.93, RMR = 0.04, RMSEA = 0.05, and TLI = 0.90. Finally, we assessed the measurement invariance across gender (male or female) using the three-factor model (Table 8). Fit indexes were adequate for each subsample (male and females) and the ΔCFI between the models was below the 0.01 cutoff. These results showed that the three-factor model do share equivalence across gender.

**TABLE 8:** Measurement invariance for gender.

Model	S-Bχ^2^	*df*	RMSEA	TLI	CFI	ΔCFI
Men	2.655.6	454	0.05	0.90	0.90	–
Women	2.637	474	0.05	0.90	0.90	–
Unconstrained	2.555.6	454	0.05	0.90	0.90	–
Measurement weights	2.637.4	474	0.05	0.89	0.90	0.000
Structural covariances	2.673.3	480	0.05	0.89	0.90	0.000

*S-Bχ^2^, Satorra-Bentler χ^2^; *df*,degrees of freedom; RMSEA, root mean square error of approximation; TLI, Tucker–Lewis fit index; CFI, comparative fit index; ΔCFI, differences in comparative fit index.*

## Discussion

Results suggest that the NAQ-R appears to be a valid instrument with suitable psychometric properties for the measurement of mobbing at the workplace. Although the version presented on this study has one more item related to physical and sexual harassment, the instrument contains internal strong structure where the first factor—related to personal harassment—is the most notable, reliable, and valid to measure harassment in accordance with the other cross-cultural validation studies (Nielsen et al., 2011).

With regard to first hypothesis, the results of the study confirm that the NAQ-R is a suitable instrument for measuring psychological harassment. All factor weights (calculated mean-variance) exceed 0.35 mean-variance as recommended (Gorsuch, 1983) and the same approach is used in further studies (e.g., Safren et al., 1998; Morrison et al., 2000).

Similar results have been found in the NAQ version of Soler et al. (2010) with an explained variance of 32.8%. The S-NAQ of León-Pérez et al. (2019b) shows an explained variance of 35.58%.

Cronbach’s alpha for the total scale (0.91) indicates similar scores compared to the original NAQ-(*N* = 4.996) indicating high internal stability (Einarsen et al., 2009). Personal harassment (Cronbach’s alpha = 0.90) is the most accurate and homogeneous underlying factor containing high internal consistency and showing discrete scores on the mobbing scale though acceptable (0.74). Physical and/or sexual related scale is less accurate as measured by Cronbach’s alpha was 0.56. As the main limitation, we find a low reliability in the third factor that shows physical and sexual harassment.

Although sexual harassment and mobbing are clearly two well-differentiated phenomena (Gimeno-Lahoz, 2004), item 23 has been included because sexual harassment is common within the process of mobbing itself, especially toward women (Lapierre et al., 2005; Cortina, 2008; Cortina and Berdahl, 2008). In addition, sexual harassment has been a defining factor in the “physical violence” dimension of mobbing (Zapf et al., 1996).

The values resulting from the third factor can be explained in two ways. The first is that victims are more hesitant to report having suffered some form of physical or sexual harm (González-Trijueque, 2007). Another possible explanation is that in countries like Spain, compared to, for example, the Latin American background, violence of a physical nature is less common due to the social concern it raises. Hence, it is not as much associated with psychological harassment at work (Dujo et al., 2019).

While analyzing concurrent validity showing clinical symptoms and burnout, we hypothesized that the scale would show negative correlations with measures on subjective health and well-being, and negative correlations with perceptions of the quality of the psychosocial work environment, including job satisfaction, commitment, and relationships with superiors and colleagues. The NAQ-R should also be moderately associated with raised levels of sick leave in addition to a strong positive association with subjective feelings of victimization (Einarsen et al., 2009).

All the aforementioned agrees with the existing literature underlining the link between harassment and the victim’s health (Leymann and Gustafsson, 1996; Niedl, 1996; Matthiesen and Einarsen, 2001, 2004; Mikkelsen and Einarsen, 2001, 2002; Borrás, 2002; Einarsen and Mikkelsen, 2003; Hogh et al., 2011; El Ghaziri et al., 2020).

Mobbing is one of the main stress factors causing devastating consequences for the employees (Moreno-Jiménez and Muñoz, 2006). The studies showed a correlation between exposure to mobbing and chronic fatigue; physical symptoms, psychosomatic, insomnia, and mental stress reactions (Dofradottir and Høgh, 2002; Einarsen and Mikkelsen, 2003; Zapf et al., 2003; Moayed et al., 2006; El Ghaziri et al., 2020). Furthermore, relevant symptoms such as musculoskeletal complaints, anxiety, irritability, and depression were reported by victims in different European countries (Einarsen and Skogstad, 1996; Niedl, 1996; Zapf et al., 1996; O’Moore et al., 1998). As well, some victims displayed a pattern of symptoms indicative of post-traumatic stress disorder (PTSD) (Björkqvist et al., 1994; Leymann and Gustafsson, 1996; Einarsen, 1999; Mikkelsen and Einarsen, 2002). According to González-Trijueque and Graña (2013), victims of mobbing were found to be more likely to show higher indicators on clinical symptoms. It must be underlined that the increased paranoid ideation rates are matching the state of hypervigilance of the victims withstanding these situations (Piñuel, 2001; Einarsen and Mikkelsen, 2003; González de Rivera and Abuin, 2006; González-Trijueque, 2010).

As for the second hypothesis, the results found in the study are similar to those obtained in the original version (Einarsen et al., 2009), in the Spanish version (González-Trijueque and Graña, 2013), and in the reduced version of Moreno-Jiménez et al. (2007). Negative correlations are observed with variables such as overall worker performance, mental and physical health, degree of health and social well-being, job satisfaction, positive correlations with somatic complaints, and desire to leave the workplace. Significant positive correlations are indicated with clinical symptoms and burnout indicators. Particularly remarkable is the correlation between personal harassment and clinical psychopathology, especially in anxiety, depression, interpersonal sensitivity, and paranoid ideation (Einarsen et al., 2009).

The same results have been found in other measures of harassment commonly used in the Spanish setting, such as the LIPT-60 (González de Rivera and Rodríguez-Abuín, 2005). In this sense, the NAQ-R adequately reflects the association in the current literature between harassment and clinical symptomatology (Matthiesen and Einarsen, 2004; Hogh et al., 2019) and burnout (Einarsen et al., 1998; Moreno-Jiménez et al., 2007).

Special mention should be given to the latest research results focusing on the correlation between mobbing and PTSD. It is generally acknowledged that mobbing does not represent a single traumatizing event but rather a systematic exposure to mainly non-physical aggression over a long-term period. It has been suggested that similar PTSD symptoms found among victims should rather be subsumed under adjustment, depressive, anxiety disorder, or simply distress that is not part of a defined psychiatric disorder category (Nielsen et al., 2015). Although exposure to psychological harassment constitutes a systematic exposure over a prolonged time period, it has been claimed that the distress many of the victims’ experience equalizes the stress associated with traumatic events (Mikkelsen and Einarsen, 2002; Matthiesen and Einarsen, 2004; Tehrani, 2004). A later meta-analysis by Nielsen et al. (2015) showed that an average of 57% of victims of bullying reported symptom scores for PTSD with an established correlation of 0.42 for exposure to harassment behaviors and PTSD symptoms.

Likewise, strong correlations on the NAQ-R were associated with psychological distress, interpersonal conflicts, deficiencies in both supervision and social support as concluded in several studies that underline correlations between the NAQ-R and a high workload pressure, negative organizational climate, negative relationship with colleagues, and low scores on organizational satisfaction (Einarsen et al., 2009).

Personal harassment factor has been found to be closely associated with clinical symptoms and especially with psychopathological scales on paranoid ideation, interpersonal sensitivity, hostility, anxiety, and depression (Table 4) as shown by González-Trijueque and Graña (2013) whereas work-related harassment has been positively associated to burnout prototypical indicator associated with emotional exhaustion. Although both are different psychosocial stressors, the mobbing phenomenon is expected to be correlated with burnout symptoms (Moreno-Jiménez et al., 2007).

As for the third hypothesis, during the early stages of the harassment process, the type of harassment that occurs may be indirect, subtle, and occasional, so that in most cases it is not detected by the victim (Nielsen et al., 2011). As the victimization process becomes more visible and direct, the nature of the harassment becomes more relevant to the victim, who begins to use all kinds of coping strategies to tackle harassment. The lack of results leads the victim to a position of vulnerability and stasis in dealing with harassment (González-Trijueque, 2007).

With regard to the findings on coping resources as stated by González-Trijueque (2007), the more unclear the harassment is, the broader is the spectrum of the employee’s coping resources, which explains the different resources the victims utilize throughout the harassment process in an attempt to stop it. All scores are correlated with both personal and work-related harassment factor. However, when there are intimidation or physical threat behaviors, victims tend to show more specific coping resources avoiding active and acceptance strategies.

It is important to highlight the lack of tolerance and cultural validation of violence in both society and Western organizational culture. While in other countries physical and sexual abuse are normalized, in most developed countries, these behaviors are unusual, which leads to a significant impact on target and results in hindering or a lack of active coping of the victim (González-Trijueque and Graña, 2013).

Finally, it can be concluded that the NAQ-R is a valid and reliable measure of exposure to harassment indicating high internal consistency and good discriminatory power. Yet, a review on the prevalence of mobbing showed that 47% of the included behavioral experience studies employed a variation of this instrument (Nielsen et al., 2010, 2011). Based on the factor structure, the NAQ-R may also be used in its one-dimension model (general/common harassment) as well as in its multi-dimension scale (personal harassment, mobbing, and physical intimidation).

After testing several underlying dimensions of the NAQ-R, findings were consistent with the data obtained (Einarsen et al., 2009). In conclusion, the confirmatory factor analysis conducted on a heterogeneous occupational sample indicates that the fit is satisfactory for the measure of exposure to mobbing on the three underlying factors in the Spanish version of the NAQ-R.

So far, the NAQ-R and other measuring instruments with similar properties have been used in both research and the context of Organizational Psychology (Notelaers and Einarsen, 2013). A needed line of research is the implementation of validity scales so that these can be applied in the forensic field with greater guarantees, taking into account the methodological needs in the field of expert assessment. The NAQ-R complies with the purpose of collecting quantitative or qualitative data that can be useful to conduct a differential diagnosis with an additional psychosocial stressor, but never to support the argument of the existing workplace harassment situation according to its outcomes (González-Trijueque and Delgado, 2011).

Although this type of tests accurately measure harassment, they are easily susceptible to manipulation (simulation) (Vilariño et al., 2020) and therefore cannot be used in the context of forensics in isolation. To meet the strict and technical criteria, they must be integrated into a multi-method and multi-source assessment system (Muñoz, 2013).

## Data Availability Statement

The datasets generated for this study are available on request to the corresponding author.

## Ethics Statement

The studies involving human participants were reviewed and approved by the Deontological Commission of the Faculty of Psychology at the Complutense University of Madrid. The patients/participants provided their written informed consent to participate in this study.

## Author Contributions

VD conducted the statistical analysis and review of the literature, wrote much of the manuscript, and interpreted the findings for this study. DG and JG encoded the data within the research process. JA was involved in the statistical analysis process (Confirmatory Factor Analysis) and monitored the drafting of the text. All authors contributed to the article and approved the submitted version.

## Conflict of Interest

The authors declare that the research was conducted in the absence of any commercial or financial relationships that could be construed as a potential conflict of interest.
